# Comprehensive amelioration of high-fat diet-induced metabolic dysfunctions through activation of the PGC-1α pathway by probiotics treatment in mice

**DOI:** 10.1371/journal.pone.0228932

**Published:** 2020-02-10

**Authors:** Jeonghyeon Kwon, Bobae Kim, Chungho Lee, Hyunchae Joung, Byoung-Kook Kim, In Suk Choi, Chang-Kee Hyun

**Affiliations:** 1 School of Life Science, Handong Global University, Pohang, Gyungbuk, South Korea; 2 Chong Kun Dang Bio Research Institute, Ansan, Gyeonggi, South Korea; State University of Rio de Janeiro, BRAZIL

## Abstract

Although the beneficial effects of probiotics in the prevention or treatment of metabolic disorders have been extensively researched, the precise mechanisms by which probiotics improve metabolic homeostasis are still not clear. Given that probiotics usually exert a comprehensive effect on multiple metabolic disorders, defining a concurrent mechanism underlying the multiple effects is critical to understand the function of probiotics. In this study, we identified the SIRT1-dependent or independent PGC-1α pathways in multiple organs that mediate the protective effects of a strain of *Lactobacillus plantarum* against high-fat diet-induced adiposity, glucose intolerance, and dyslipidemia. *L*. *plantarum* treatment significantly enhanced the expression of SIRT1, PPARα, and PGC-1α in the liver and adipose tissues under HFD-fed condition. *L*. *plantarum* treated mice also exhibited significantly increased expressions of genes involved in bile acid synthesis and reverse cholesterol transport in the liver, browning and thermogenesis of adipose tissue, and fatty acid oxidation in the liver and adipose tissue. Additionally, *L*. *plantarum* treatment significantly upregulated the expressions of adiponectin in adipose tissue, irisin in skeletal muscle and subcutaneous adipose tissue (SAT), and FGF21 in SAT. These beneficial changes were associated with a significantly improved HFD-induced alteration of gut microbiota. Our findings suggest that the PGC-1α-mediated pathway could be regarded as a potential target in the development of probiotics-based therapies for the prevention and treatment of metabolic disorders.

## Introduction

Over the past decade, the gut microbiota has emerged as an important regulator of host metabolism and the alterations in its composition have been known to contribute to the development of obesity and its complications including insulin resistance, nonalcoholic fatty liver disease (NAFLD), hypercholesterolemia, and hyperlipidemia [1]. Modulation of the gut microbiota by probiotics thereby has been considered as a new approach for the treatment of metabolic disorders [2,3]. Several studies have described the ameliorating effects of specific probiotic strains, in particular those of the *Lactobacillus* and *Bifidobacterium*, on the characteristics of metabolic syndrome, such as preventing weight gain and fat deposition, improving glucose intolerance and dyslipidemia, and modulating chronic inflammation [4–6]. However, most studies have focused on specific effects of particular disorders, therefore little is known about the comprehensive impacts of probiotics on host metabolic dysfunctions in metabolic disorders. In this study, we aimed to characterize the probiotic properties of a strain of *Lactobacillus plantarum* and investigate the mechanisms underlying its comprehensive effects on several metabolic parameters associated with metabolic disorders.

Sirtuin 1 (SIRT1) plays a critical role in metabolic homeostasis by activating target proteins in various tissues including the liver, adipose tissue, and skeletal muscle [7]. SIRT1 enhances PPARα activity by deacetylating the co-activator PGC-1α, which stimulates the expression of genes involved in mitochondrial biogenesis and fatty acid oxidation [8]. Furthermore, SIRT1 promotes a “fasting-like” status favoring hepatic cholesterol clearance through the activation of CYP7A1, a rate limiting enzyme in bile acid synthesis, and also reverse cholesterol transport (RCT) through upregulating ABCA1 and SR-B1, two primary high-density lipoprotein (HDL)-cholesterol efflux transporters [7, 9]. Despite the critical roles of SIRT1 in metabolic regulation, there have been no studies suggesting that the beneficial effects of probiotics on risk factors for metabolic disorders are mediated by the positive modulation of SIRT1/PGC-1α pathway.

Adiponectin, an adipocyte-derived hormone, is known to play an important role in the glucose and lipid metabolism, improving insulin sensitivity and lipid profiles through multiple mechanisms in a variety of tissues [10]. Adiponectin signaling through its receptors AdipoR1 and AdipoR2 stimulates AMPK that subsequently activates PGC-1α by direct phosphorylation or SIRT1-mediated deacetylation, leading to increased fatty acid oxidation, resulting in reduced fat accumulation in the liver, skeletal muscle, and adipose tissue [11–13]. Recent studies have shown that irisin and fibroblast growth factor 21 (FGF21) have significant roles in the regulation of energy metabolism through stimulating white adipose tissue (WAT) browning and potentiating brown adipose tissue (BAT) function via PGC-1α-dependent pathways [14].

Irisin, an adipomyokine, upregulates the expression of browning-associated genes and UCP1 protein in adipose tissue, resulting in enhanced fatty acid oxidation and thermogenesis, while suppressing adipogenesis [15,16]. Adipose-derived FGF21 also increases the expression of UCP1 and other thermogenic genes in adipose tissue by enhancing PGC-1α expression [17,18]. Browning of WAT and thermogenesis in BAT are major contributors to energy expenditure, which have been considered as effective strategies for the treatment of metabolic disorders [19]. It also has been proposed by several studies that WAT browning and BAT thermogenesis could be regulated through modulation of microbiota and their derived metabolites [20]. However, there have been few studies describing how probiotic modulation of gut microbiota influences adipose tissue metabolism, and the mechanisms through which probiotics may exert enhancing effects on adipose tissue browning and thermogenesis are poorly understood.

In this study, we explored the mechanisms underlying the probiotic effect of a stain of *L*. *plantarum* that exerts multiple beneficial effects, including reducing adiposity and improving glucose intolerance and dyslipidemia in high-fat diet (HFD)-induced obese mice. We especially focused on the evaluation of favorable changes in glucose and lipid metabolism in the liver and adipose tissue, cholesterol and bile acid metabolism in the liver, browning and thermogenesis in adipose tissue resulted from probiotic supplementation, and investigated the molecular mechanisms underlying the changes. Our results demonstrate that some important metabolic homeostasis regulators such as adiponectin, irisin, and FGF21 contribute to the probiotic effect on host metabolism through activation of SIRT1/PGC-1α dependent pathway, which is a concurrent mechanism to describe the diverse effects of probiotics observed in several metabolic tissues. Given the significant role of inter-organ metabolic crosstalk for whole body energy homeostasis, defining a concurrent mechanism underlying the multiple effects of probiotics is critical to broaden the understanding of the impact of probiotics on host metabolism. Our findings provide evidence to better understand the role of probiotics in treating metabolic abnormalities and suggest that the treatment of *L*. *plantarum* probiotics could be a therapeutic approach to achieve comprehensive improvement of metabolic disorders.

## Materials and methods

### Bacterial strain and culture conditions

*L*. *plantarum* Q180 strain was kindly provided by Chong Kun Dang Bio Co. (Ansan, Korea), which was originated from the fecal sample of healthy adult volunteer [21]. The strain was grown in MRS broth (Difco Laboratories INC., Franklin Lakes, NJ) at 37°C, lyophilized, and then stored at -70°C until further use. For the administration to mice, cell suspensions were daily prepared by suspending the strain in PBS and adjusting the viable count to 1 × 10^9^ or 1 × 10^10^ CFU/mL.

### Animal experiments

Six-week-old C57BL/6L male mice purchased from Central Lab. Animal Inc. (Seoul, Korea) were housed at 22 ± 1°C and 45 ± 10% humidity, on a 12 h light/dark cycle. After 2 weeks of adaption, mice were divided into five experimental groups (n = 10 per groups) each receiving different treatments; normal diet (ND)-fed control, high-fat diet (HFD)-fed control, HFD-fed *Lactobacillus rhamnosus* GG (LGG, a probiotic control strain)-treated (1x10^9^ CFU/day), and HFD-fed low-dose (1x10^9^ CFU/day) *L*. *plantarum*–treated (LDLP) and high-dose (1x10^10^ CFU/day) *L*. *plantarum*–treated (HDLP) groups. Each group was fed with ND (10%kcal from fat, D12450J, Research Diets Inc., New Brunswick, NJ) or HFD (60%kcal from fat, D12492) for 1 week, and during following 12 weeks, mice received oral gavage with 100 μL PBS or a daily dose of probiotics (LGG or *L*. *plantarum*) with ND or HFD feeding. On the last day of the experiment, mice were sacrificed and tissue samples were harvested as previously described [22]. All animal experiments were performed in accordance with protocols approved by the Committee on the Ethics of Animal Experiments of the Handong Global University (Permit number: 20190424–009)

### Serum and hepatic lipid analyses

For measure the concentration of total cholesterol, blood samples were obtained from tail vein in 4 h fasted mice after 11 weeks of LGG or *L*. *plantarum* treatment, and total cholesterol levels were measured using Accutrend Plus meter (Roche Diagnostics Ltd., Basel, Switzerland). For ELISA and HDL-cholesterol assay, blood samples were obtained from heart in 4 h fasted mice after 12 weeks of LGG or *L*. *plantarum* treatment. Serum samples were collected, centrifuged, and stored at −70°C until use. Levels of serum insulin and HDL-cholesterol were analyzed using Ultra Sensitive Mouse Insulin sandwich ELISA kit (Morinaga Inst. Biol. Sci., Yokohama, Japan) and HDL-cholesterol assay kit (Asan Pharm., Seoul, Korea), respectively. Triglycerides (TG) levels in the liver were quantified by the colorimetric assay as described previously [22]. Briefly, the liver was homogenized in chloroform/methanol (2:1) solution using a hand-held homogenizer (IKA, Stufen, Germany), and then incubated for 2 h at room temperature. After adding 1M H_2_SO_4_, the lysate was centrifuged at 2,000 rpm for 20 min. The separated bottom layer containing TG and phospholipids was mixed with 1% Triton X-100/chloroform solution and dried overnight at room temperature. Dried samples were resuspended in water, mixed with TG assay buffer (TG-S assay kit, Asan Pharm.) to measure TG using SPECTROstar Nano (BMG Labtech, Offenburg, Germany).

### Glucose and lipid tolerance test

After 10 weeks of LGG or *L*. *plantarum* treatment, mice were fasted for 4 h, with free access to water, prior to the test. Glucose was injected intraperitoneally at concentration of 2 g/kg body weight, and the glucose levels in blood samples from tail bleeds were measured using GlucoDr auto AGM-4000 (Allmedicus, Anyang, Korea) at baseline and 15, 30, 60, 90 and 120 min after glucose injection. After 11 weeks of LGG or *L*. *plantarum* treatment, followed by 4 h fasting, mice were orally administered with olive oil at a dose of 5 ml/kg, and the TG levels in tail blood samples were measured with Accutrend Plus meter at 0, 60, 120, 180 and 240 min after oil injection.

### Histological analysis

Tissue samples of the liver, quadriceps skeletal muscle, subcutaneous adipose tissues (SAT), mesenteric adipose tissue (MAT), and interscapular brown adipose tissue (BAT) were fixed, H&E stained and examined by light microscopy as described previously [22]. Briefly, 5-μm-thick microtome sections of 10% v/v formalin/PBS-fixed, paraffin-embedded tissues were prepared, and stained with hematoxylin and eosin. Microscope images were obtained at 200X, and the areas of adipocytes were measured using ImageJ Adiposft software [23].

### Real-time RT PCR

Total RNA extraction, reverse transcription and quantitative PCR were conducted as described previously [22]. Quantification of gene transcripts for cholesterol 7 alpha-hydroxylase (CYP7A1), CYP7B1, CYP27A1, CYP8B1, bile salt export pump (BSEP), β-klotho, fibroblast growth factor receptor 1c (FGFR1c), FGFR4, G protein-coupled bile acid receptor (TGR5), farnesoid X receptor (FXR), FGF15, HMG-CoA reductase (HMGCR), HMG-CoA synthase (HMGCS), scavenger receptor class B type 1 (SR-B1), ATP-binding cassette subfamily G member 5 (ABCG5), ABCG8, lecithin: cholesterol acyltransferase (LCAT), apolipoprotein A1 (ApoA1), liver X receptor α (LXRα), adiponectin, adiponectin receptor 1 (AdipoR1), AdipoR2, SIRT1, irisin, fibroblast growth factor 21 (FGF21), peroxisome proliferator-activated receptor α (PPARα), PPARγ coactivator 1α (PGC-1α), NADH-ubiquinone oxidoreductase chain 5 (ND5), Prdm16, Dio2, Cidea, Elov13, uncoupling protein 1 (UCP1), carnitine palmitoyltransferase 1 (CPT1), acyl-CoA oxidase 1 (ACOX1), and medium-chain acyl-coenzyme A dehydrogenase (MCAD), diacylglycerol acyltransferase 1 (DGAT1), DGAT2, PPARγ, sterol-regulatory element binding protein 1c (SREBP1c), acetyl-CoA carboxylase (ACC), fatty acid synthase (FAS), glycerol-3-phosphate acyltransferase (GPAT), stearoyl-CoA desaturase 1 (SCD1), acidic ribosomal phosphoprotein (Arbp), and β-actin was performed using gene-specific primers. Primer-BLAST tool used for design target-specific primers (https://www.ncbi.nlm.nih.gov/tools/primer-blast/). Primer sequences are available in S2 Table. Results were presented as means ± S.D. Results were presented as means ± S.D. normalized to expression of Arbp or β-actin using the ΔΔ Ct method, in which the HFD-fed control (HFD+PBS) group was used as the reference group.

### Western blot analysis

Western blot analysis was performed as described previously [22]. Antibodies against total AMPK (#2532, Cell signaling technology, Beverly, MA), phospho (Thr172) AMPK (#2531) were used as primary antibodies, followed by anti- rabbit IgG-HRP conjugated secondary antibody (#7074).

### Gut microbiota analysis using NGS

Metagenomic analysis was performed following the protocol for 16S Metagenomic Sequencing Library Preparation Part # 15044223 Rev. B (Illumina platform) [24]. Briefly, DNA samples extracted from the fecal sample were randomly cut and ligated 5’ and 3’ adapters to both ends of the fragments to make a library construction. Each fragment is bridge amplified in a flow cell making clonal clusters. Paired-end sequencing of DNA samples were carried out on the Illumina sequencer to generate raw images. The base-calling software called Real Time Analysis was used to process the raw data.

### Statistical analyses

The experimental results were presented as means ± S.D for 6–10 mice in each group. Statistical analyses were performed using GraphPad Prism software (GraphPad, version 8, San Diego, CA). For the analysis of data of body weight, tissue weight, serum TG and cholesterol, one-way or two-way analysis of variance (ANOVA) with Dunnett’s multiple comparison test was used to determine statistical significance. *P* values < 0.0332 were considered as statistically significant. For the analysis of data from glucose tolerance test, lipid tolerance test, serum insulin analysis, real-time PCR, and western blotting, group means were compared using a student’s two-tailed t-test. *P* values < 0.05 were considered as statistically significant.

## Results

### *L*. *plantarum* treatment reduces adiposity and improves glucose and lipid intolerance in HFD-fed mice

**Fig 1:**
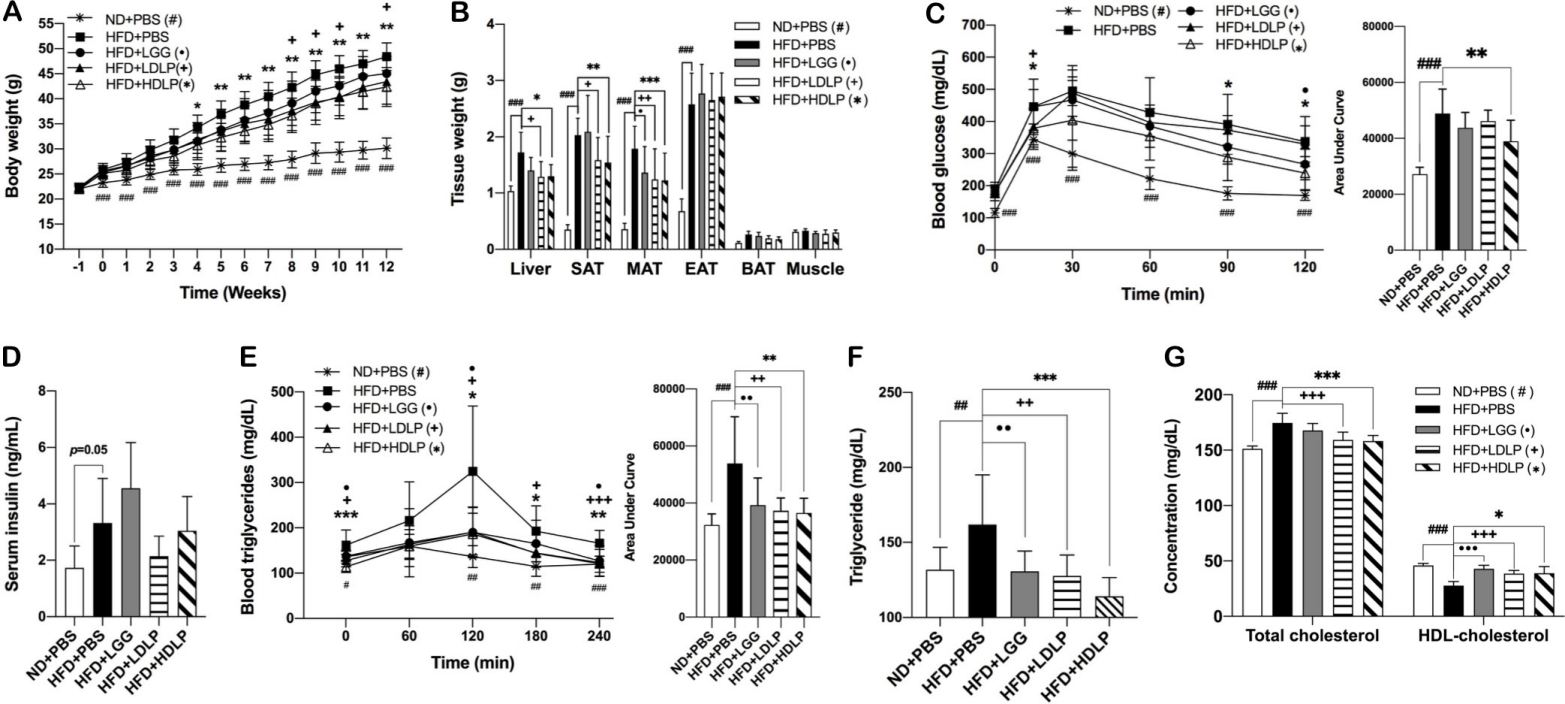
*L*. *plantarum* treatment reduces adiposity and improves glucose and lipid intolerance in HFD-fed mice. (A) Body weight changes for 13 weeks of HFD feeding with LGG or *L*. *plantarum* treatment for latter 12 weeks (n = 9–10). (B) Changes in tissue weight after 12 weeks of LGG or *L*. *plantarum* treatment (n = 9–10). (C) Serum concentration of insulin after 12 weeks of LGG or *L*. *plantarum* treatment quantified by ELISA (n = 6). (D and E) Intraperitoneal glucose tolerance test and oral lipid tolerance test in mice at 10 and 11 weeks of LGG or *L*. *plantarum* treatment, respectively (n = 9). (F and G) Blood levels of TG, total cholesterol, and HDL-cholesterol after 11 weeks of LGG or *L*. *plantarum* treatment. Statistical significance was determined using two-way ANOVA with (Fig 1A and 1B) or ordinary one-way ANOVA (Fig 1F and 1G) with Dunnett’s comparison test. ^#, •, +, *^p < 0.0332, ^##, ••, ++, **^ *p* < 0.0021, ^###, •••, +++, ***^ *p* < 0.0002. Student’s two-tailed t-test was used for analysis of differences between experimental groups (Fig 1C, 1D, and 1E). ^#, •, +, *^p < 0.05, ^##, ••, ++, **^ *p* < 0.01, ^###, •••, +++, ***^ *p* < 0.001. ND: normal chow diet, HFD: high-fat diet, PBS: phosphate buffered saline, LDLP and HDLP: low- and high-dose of *L*. *plantarum*, SAT: subcutaneous adipose tissue, MAT: mesenteric adipose tissue, BAT: brown adipose tissue.

*L*. *plantarum* treatment significantly reduced HFD-induced body weight gain, which was parallel to significant decreases in the weight of tissues including the liver, subcutaneous (SAT), and mesenteric adipose tissue (MAT) as compared to non-treated HFD-fed control mice (Fig 1A and 1B). There was a noticeable improvement in glucose tolerance of high-dose *L*. *plantarum* treated mice, with no significant change of serum insulin levels, compared to their HFD-fed controls (Fig 1C and 1D). *L*. *plantarum* treatment also significantly improved lipid tolerance in both low- and high-dose treated mice (Fig 1E). Furthermore, serum lipid profiles showed a significant reduction in TG and total cholesterol and increase in HDL-cholesterol in both low-dose and high-dose *L*. *plantarum* treated mice compared to those of HFD-fed controls (Fig 1F and 1G). Together, these results indicated that the treatment of *L*. *plantarum* improved HFD-induced glucose intolerance and blood lipid abnormalities.

### *L*. *plantarum* treatment improves bile acid synthesis and reverse cholesterol transport via SIRT1-PGC-1α pathway

**Fig 2:**
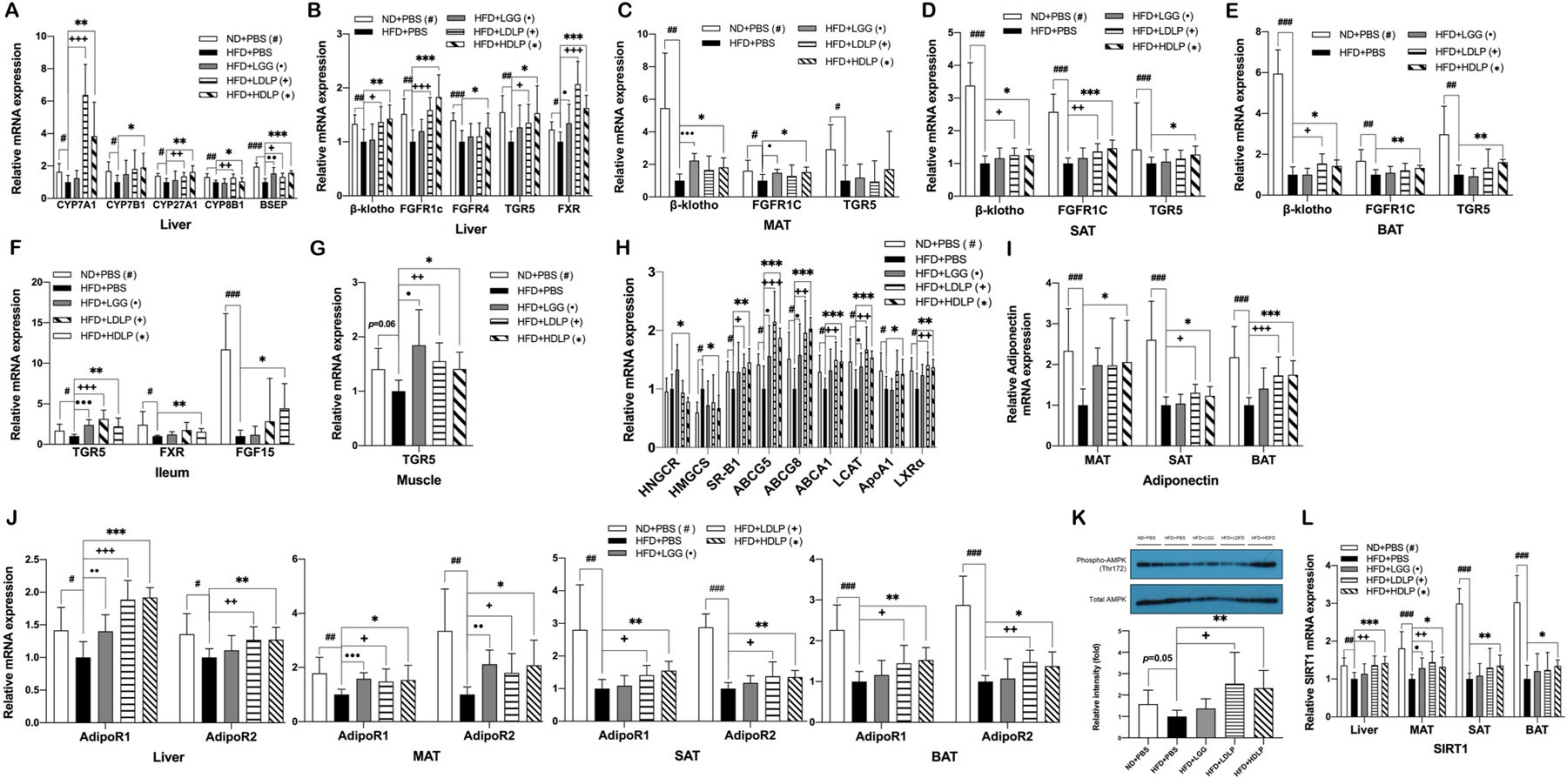
*L*. *plantarum* treatment improves bile acid synthesis and reverse cholesterol transport. Effects of *L*. *plantarum* treatment on mRNA expression of (A) genes involved in bile acid synthesis in the liver, (B-G) bile acid and FGF15 receptors in the liver, MAT, SAT, BAT, ileum, and skeletal muscle, respectively, (H) genes related to RCT in the liver, (I) adiponectin in adipose tissue, (J) adiponectin receptors in the liver and adipose tissue, and (L) SIRT1 in the liver and adipose tissue. Gene expression were analyzed by real-time PCR using gene-specific primers. The liver and adipose tissue genes are normalized to expression of β-actin, skeletal muscle and ileum genes are normalized to expression of Arbp. Data present mean ± SD for 7~8 mice in each group. (K) AMPK phosphorylation in the liver detected by SDS-PAGE-immunoblotting. Data present mean ± SD. of fold changes in blot intensity compare with HFD+PBS group. Student’s two-tailed t-test was used for analysis of differences between groups. ^#, •, +, *^p < 0.05, ^##, ••, ++, **^ *p* < 0.01, ^###, •••, +++, ***^ *p* < 0.001.

To examine how *L*. *plantarum* attenuated the impaired glucose and lipid tolerance of HFD-fed mice and improved their serum lipid profile, we first assessed the molecular changes in peripheral metabolic tissues such as the liver, adipose tissue (MAT, SAT, and BAT), skeletal muscle, and ileum. Given the fact that bile acids play an essential role in maintaining TG and cholesterol homeostasis, we tested the expression of genes involved in bile acid synthesis. We found that, in the liver of *L*. *plantarum* treated mice, the expression of bile acid synthetic genes such as CYP7A1, CYP7B1, CYP27A1 and CYP8B1 were significantly increased, with a commensurate increase in gene expression of bile acid transporter BSEP (Fig 2A). The expression level of ileal enterocyte-derived hormone FGF15 was also significantly higher in ileum of *L*. *plantarum* treated mice than that of non-treated HFD-fed controls (Fig 2F). In addition, the expressions of bile acid receptor TRG5 and receptors for FGF15 (β-klotho/FGFR4 or β-klotho/FGFR1c) were also increased in the liver, SAT, ileum, and skeletal muscle of *L*. *plantarum* treated mice (Fig 2B–2G).

Along with increased expression of bile acid synthetic genes, *L*. *plantarum* treatment also exerted beneficial effects on cholesterol metabolism through suppression of cholesterol synthesis and improvement of RCT, a process by which excess cholesterol from peripheral tissues returns to the liver. The gene expression of cholesterol synthetic enzymes, HMGCR and HMGRS, was significantly reduced, and the expression of RCT related genes including SR-B1, ABCG5, ABCG8, LCAT, ApoA1, and LXRα was significantly increased in the liver of *L*. *plantarum* treated mice (Fig 2H).

Adiponectin is one of the key adipokines that controls energy metabolic homeostasis in peripheral tissues [10]; therefore, we tested whether there is an impact of *L*. *plantarum* treatment on adiponectin expression in adipose tissue. In all three adipose tissues, including MAT, SAT and BAT, of *L*. *plantarum* treated groups, the expression of adiponectin was significantly increased compared to non-treated HFD-fed control group (Fig 2I), which was accompanied by upregulation of adiponectin receptors, AdipoR1 and AdipoR2, in the liver, MAT, SAT, and BAT (Fig 2J). The augmented expression of adiponectin and its receptors resulted in an enhanced phosphorylation of AMPK in the liver (Fig 2K), indicating that the beneficial lipid-metabolic effect of *L*. *plantarum* was mediated, at least in part, by activation of adiponectin-AMPK pathway. Since the AMPK-SIRT1 axis reportedly plays important roles in regulating adiponectin signaling and in the lipid-lowering action of adiponectin [11, 25, 26], we further examined the expression of SIRT1. It was observed that its expression level was significantly higher in the liver, MAT, SAT, and BAT of *L*. *plantarum* treated mice than that of non-treated HFD controls (Fig 2L), indicating that enhanced SIRT1 signaling also contributed to the protective action of *L*. *plantarum* against HFD-induced disturbance of TG and cholesterol homeostasis. All these enhancing effects of *L*. *plantarum* on expression of adiponectin, SIRT1, bile acid synthetic genes and TGR5 were not observed in LGG-treated mice (Fig 2).

### *L*. *plantarum* treatment enhances WAT browning and BAT thermogenesis

**Fig 3:**
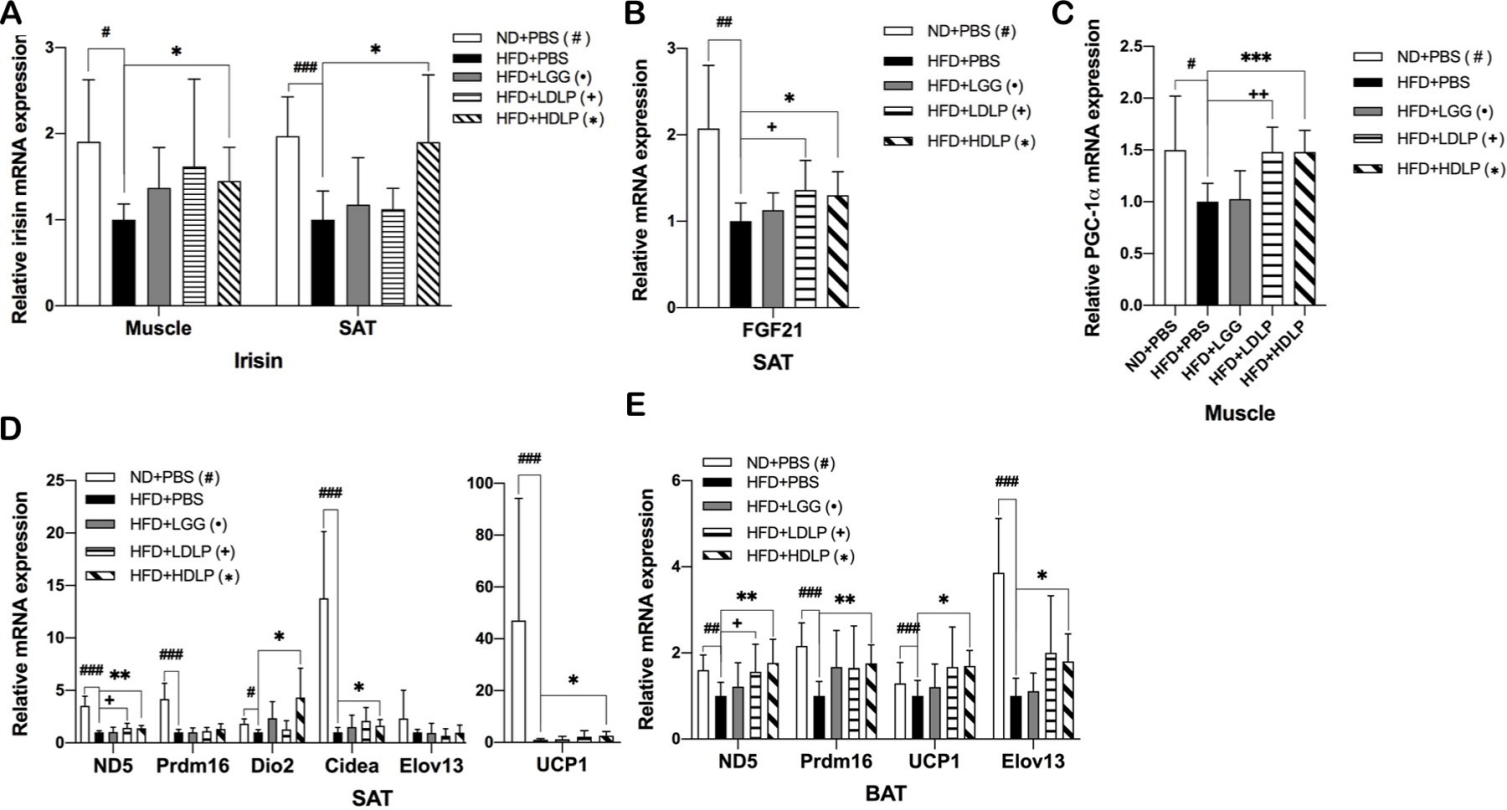
*L*. *plantarum* treatment induces browning and non-shivering thermogenesis. Effects of *L*. *plantarum* treatment on mRNA expression of (A) irisin in skeletal muscle, (B) FGF21 in SAT, (C) PGC-1α in skeletal muscle, and (D-E) genes involved in browning and thermogenesis in SAT and BAT. Adipose tissue genes are normalized to expression of β-actin, skeletal muscle genes are normalized to expression of Arbp. Data present mean ± SD for 7~8 mice in each group. Student’s two-tailed t-test was used for analysis of differences between groups. ^#, •, +, *^p < 0.05, ^##, ••, ++, **^ *p* < 0.01, ^###, •••, +++, ***^ *p* < 0.001.

**Fig 4:**
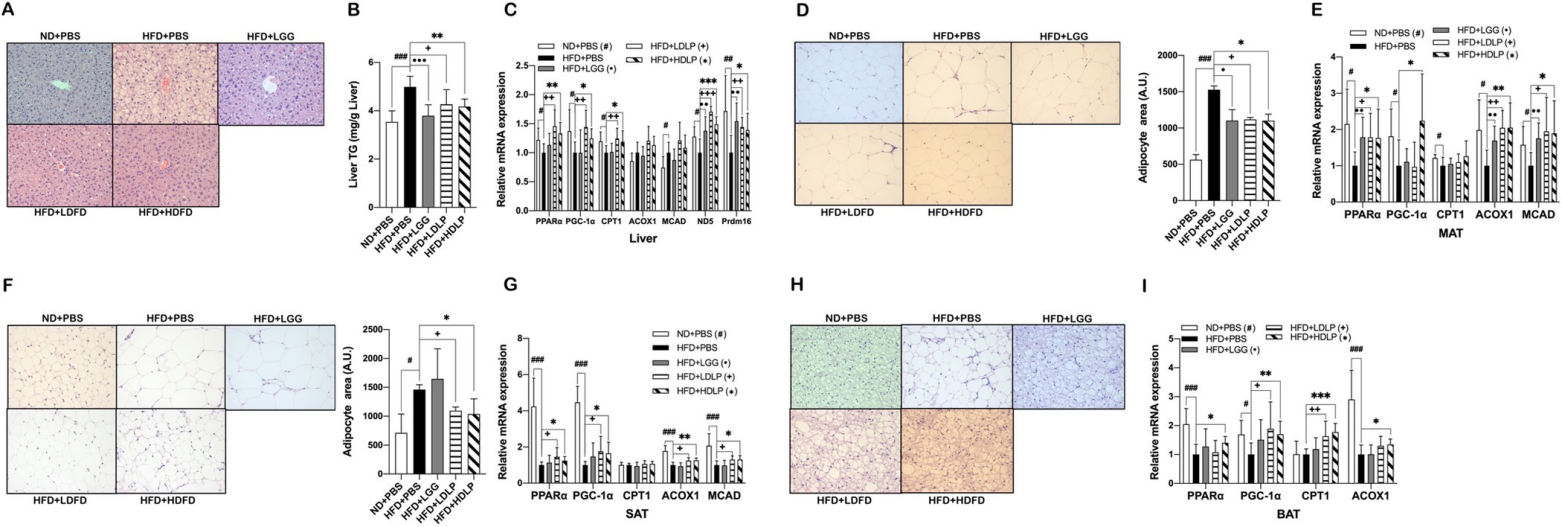
*L*. *plantarum* treatment improves lipid oxidation in the liver and white adipose tissue. (A) Changes in hepatic adiposity after 12 weeks of *L*. *plantarum* treatment. Shown are representative photomicrographs (200X) of the liver sections stained with hematoxylin and eosin (n = 3). (B) Effect of *L*. *plantarum* treatment on the liver TG accumulation (n = 8). (C, E, G, and I) Effect of *L*. *plantarum* treatment on mRNA expression related to fatty acid oxidation in the liver, MAT, SAT, and BAT, respectively. (D, F, and H) Changes in adipocyte size in MAT, SAT, and BAT, respectively with representative photomicrographs (n = 3). All genes are normalized to expression of β-actin. mRNA data present mean ± SD for 7~8 mice in each group. Student’s two-tailed t-test was used for analysis of differences between groups. ^#, •, +, *^p < 0.05, ^##, ••, ++, **^ *p* < 0.01, ^###, •••, +++, ***^ *p* < 0.001.

Induction of WAT browning and thermogenesis and potentiation of BAT thermogenesis are stimulated by hormones such as irisin [15] and FGF21 [17], which are mediated by SIRT1-PGC-1α pathway. Bile acids also appear to play endocrine roles through their receptor TGR5 that promotes energy expenditure by increasing WAT browning [27] and BAT activity [28]. To determine whether the improvement of HFD-induced dyslipidemic profile in *L*. *plantarum* treated mice was a consequence of increased energy expenditure through non-shivering thermogenesis via BAT activation and WAT browning, we examined the changes in expression of genes associated with browning and thermogenesis. The mRNA expression levels of irisin in skeletal muscle and SAT, and of FGF21 in SAT of *L*. *plantarum* treated mice, but not LGG-treated mice, were observed to be significantly higher than those of non-treated HFD-fed control mice (Fig 3A and 3B), which was associated with upregulated expression of PGC-1α in skeletal muscle and SAT (Figs 3C and 4G). Moreover, we observed that mice treated with *L*. *plantarum* had significantly increased expressions of adipocyte-specific thermogenic genes such as ND5, Prdm16, Cidea, Elov13, and UCP1 in SAT and BAT (Fig 3D and 3E), indicating an enhancement in browning of SAT and thermogenic capacity of SAT/BAT, which was not observed in LGG treated mice.

### *L*. *plantarum* treatment improves lipid oxidation in the liver and adipose tissue

Next, we evaluated the protective effects of *L*. *plantarum* against HFD-induced fat accumulation in peripheral tissues, and assessed the changes in expression of genes involved in lipogenesis and lipid oxidation. Fat deposition in the liver was significantly reduced (Fig 4A and 4B) and adipocyte sizes in MAT, SAT, and BAT were all noticeably decreased in *L*. *plantarum* treated mice (Fig 4D, 4F and 4H). Consistent with these histological data showing decreased fat accumulation, the mRNA expression levels of PPARα, PGC-1α, and lipid oxidative genes including CPT1, ACOX1, and MCAD were markedly increased in the liver and adipose tissues of *L*. *plantarum* treated mice compared to non-treated HFD-fed controls (Fig 4C, 4E, 4G and 4I). However, there was no significant difference in the expression levels of lipogenic genes including DGAT, PPARγ, SREBP1c, ACC, FAS, and GPAT, between *L*. *plantarum* treated and non-treated HFD-fed groups (S1 Fig). The changes in expressions of lipid oxidation-related genes induced by *L*. *plantarum* treatment were not observed in LGG treated mice.

### *L*. *plantarum* treatment reverses HFD-induced gut microbiota alteration

**Fig 5:**
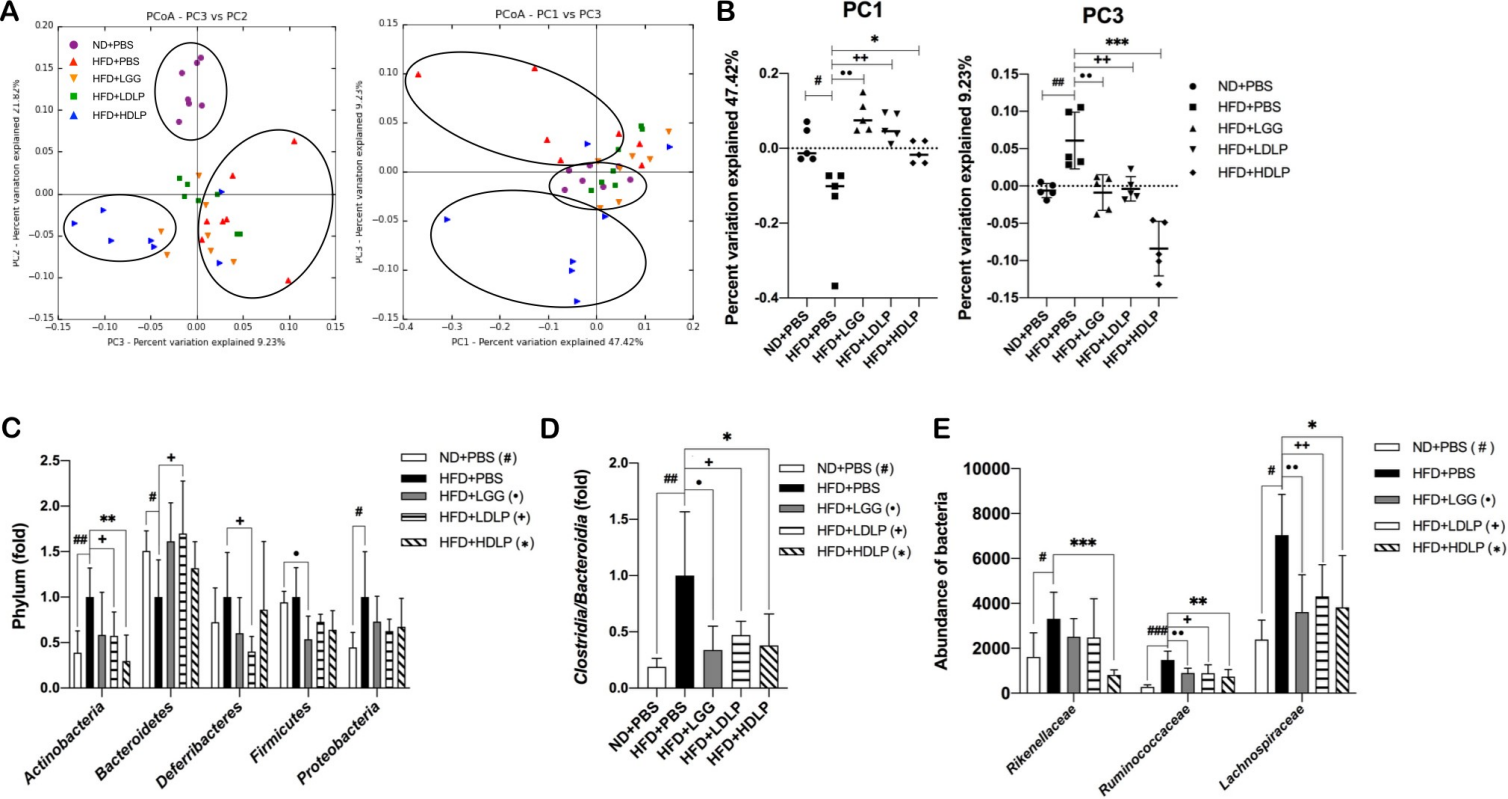
*L*. *plantarum* treatment modulate the gut microbial population. (A) Principal coordinated analysis (PCoA) plots generated from weighted UniFrac distance metrics (n = 7). (B) The value of PC1 and PC3 from weighted UniFrac distance metrics matrix (n = 5). Each dot represents one mouse. (C) Relative abundance of phylum level bacteria in fecal sample (n = 6–7). (D) The ratio of *Clostridia* to *Bacteroidia* classes (n = 6–7). (E) Relative abundance of family level bacteria in fecal sample (n = 6–7). Data present mean ± SD. Student’s two-tailed t-test was used for analysis of differences between groups. ^#, •, +, *^p < 0.05, ^##, ••, ++, **^ *p* < 0.01, ^###, •••, +++, ***^ *p* < 0.001.

To determine whether the beneficial metabolic effects of *L*. *plantarum* treatment mentioned above are associated with modulation of gut microbiota, we compared the microbiota profiles of fecal samples using bacterial 16S rRNA gene analysis. Principal coordinate analysis (PCoA) of beta-diversity present in the fecal bacterial communities revealed significant separation between high-dose *L*. *plantarum*, but not LGG or low-dose *L*. *plantarum*, treated mice and non-treated HFD-fed control mice. (Fig 5A and 5B). We also found differences in the relative abundance of specific bacterial taxa in gut microbiota associated with *L*. *plantarum* treatment. At the phylum level, while the HFD-induced microbiota dysbiosis was characterized by increased Actinobacteria and decreased Bacteroidetes compared to ND-fed controls, the abundance of Actinobacteria was significantly lower in both low- and high-dose *L*. *plantarum* treated groups (in the case of Bacteroidetes, higher abundance in low-dose, but not high-dose, *L*. *plantarum* treated group) than that of non-treated HFD-fed controls (Fig 5C). Furthermore, at the class level, the ratio of the Firmicutes-associated class *Clostridia* to the Bacteroidetes-associated class *Bacteroidia* was significantly reduced in *L*. *plantarum* treated mice when compared to non-treated HFD-fed controls (Fig 5D). Additionally at the family level, the abundance of *Rikenellaceae*, *Ruminococcaceae*, and *Lachnospiraceae* was significantly decreased in *L*. *plantarum* treated mice compared to non-treated HFD-fed control mice (Fig 5E). Taken together, these data indicated that the treatment of *L*. *plantarum* was able to reverse HFD-induced gut microbiota dysbiosis, which might contribute to improvement of metabolic dysfunctions in HFD-induced obese mice.

## Discussion

The goal of this study was to explore the mechanisms underlying the beneficial metabolic actions of a strain of *L*. *plantarum* having a comprehensive effect that simultaneously alleviates multiple metabolic disturbances. The *L*. *plantarum* strain used in this study exerted a wide-range of favorable metabolic effects, including improvements in adiposity, glucose intolerance, hepatic steatosis, and dyslipidemia in HFD-induced obese mice (Figs 1 and 4). Given the fact that the crosstalk between metabolic organs plays a central role in maintaining energy homeostasis, we hypothesized a concurrent mechanism that could underlie these effects, and found that *L*. *plantarum* treatment activated the PGC-1α-mediated pathways in the liver and adipose tissues of HFD-fed mice, which led to improvements in bile acid synthesis, RCT, lipid oxidation, WAT browning, and BAT thermogenesis.

SIRT1, referred to as a master metabolic regulator, is known to protect the functions of liver and adipose tissue against metabolic dysregulation [29], and increased expression of SIRT1 is associated with improved glucose and lipid metabolism [30]. For example, SIRT1 plays a critical role in fatty acid and cholesterol metabolism in the liver via deacetylation of PGC-1α [8]. In this study, we observed that SIRT1 expression was significantly upregulated by *L*. *plantarum* treatment in the liver and adipose tissues of HFD-fed mice (Fig 2L), which might lead to improvements of lipid metabolic dysfunctions in those tissues. Bile acid synthesis and RCT were increased together with a reduction in cholesterol synthesis in the liver (Fig 2A and 2H), and fatty acid oxidation was enhanced concomitantly in all tissues tested, including the liver, MAT, SAT, and BAT (Fig 3).

Bile acids, apart from their well-known functions in cholesterol homeostasis and lipid digestion, play an important role in regulating glucose and lipid homeostasis as signaling molecules and metabolic regulators [31]. It has been reported that increase in circulating bile acids and consequent activation of bile acid signaling can improve metabolic disorders, reducing adiposity, enhancing thermogenic capacity, and improving glucose and lipid homeostasis [32,33]. Bile acid synthesis and transport are also important for maintaining cholesterol homeostasis and preventing fat and cholesterol accumulation in the liver and other organs [34]. Bile acids are synthesized from cholesterol in the liver through two pathways: the classic or alternative pathway, controlled by the rate-limiting enzymes CYP7A1 and CYP27A1, respectively [34]. The efflux processes of bile acids into bile are mainly mediated by BSEP located in the canalicular membrane of hepatocytes. In the ileum, bile acids are reabsorbed into enterocytes and activate FXR to induce FGF15 (the ortholog of human FGF19), which is circulated to hepatocytes to activate FGFR4/β-klotho complex [34]. Bile acids also activate the signaling through their receptor, TGR5, which is expressed in many tissues including the liver (hepatic macrophages but not hepatocytes), intestine, skeletal muscle, and adipose tissues, leading to enhancement of energy expenditure, protection against fat accumulation, and improvement of insulin sensitivity [35].

However, little information is available on the mechanism how the administration of probiotics affects bile acid metabolism and thereby lowers blood cholesterol and TG. Here, we present evidence that *L*. *plantarum* probiotics can modulate bile acid metabolism through regulating the synthesis and secretion of bile acids. *L*. *plantarum* treatment promoted both the classical and alternative bile acid synthetic pathways as indicated by increased expression of CYP7A1, CYP7B1, CYP27A1, and CYP8B1 in the liver of HFD-fed mice (Fig 2A). Increased bile acid efflux by *L*. *plantarum* treatment was also evident from the elevated expression of BSEP (Fig 2A). These modulations might play a role on the reduction in circulating cholesterol and TG in *L*. *plantarum* treated mice. We also found that the increased bile acid synthesis led to an increase in TGR5 expression in the liver, SAT, BAT, ileum, and skeletal muscle (Fig 2E–2G), which might result in enhanced browning and thermogenesis in SAT and BAT and reduced hepatic fat accumulation. Furthermore, receptors (β-klotho/FGFR4 or β-klotho/FGFR1c) for FGF15, a hormone induced by bile acid-activated FXR in ileal enterocytes, were also upregulated in the same tissues by *L*. *plantarum* treatment (Fig 2B–2G). FGF15/19 has been known that is able to increase metabolic rate and BAT-mediated energy expenditure concurrently with an increase in fatty acid oxidation, leading to reduced hepatic steatosis and enhanced insulin sensitivity [36]. Our data suggest that, in addition to the direct effect of bile acids mediated through activation of TGR5, the enhanced production of FGF15 in response to bile acid absorption in enterocytes, might also contribute to the *L*. *plantarum* treatment-mediated attenuation of HFD-induced metabolic abnormalities.

To maintain cholesterol homeostasis, the intricate network of cholesterol-related processes such as cholesterol biosynthesis, intestinal absorption, lipoprotein release into the blood, and transport to the liver, must be tightly regulated [37]. RCT is the major route for removal of excess cholesterol from peripheral tissues and its transport to the liver, by which HDL protects against atherosclerotic cardiovascular disease [37]. In RCT, excess cholesterol from macrophage foam cells is transferred and carried by HDL to the liver for excretion as bile acids or free cholesterol into the feces, including ApoA1-activated LCAT-mediated HDL maturation, HDL cholesterol uptake via SR-BI, and biliary cholesterol secretion by ABCG5 and ABCG8 [38]. Our results showed an increased hepatic expression of SR-B1, ABCG5, ABCG8, LCAT, and ApoA1 in *L*. *plantarum* treated mice (Fig 2H), from which it is indicated that *L*. *plantarum* treatment contributes to prevent elevated cholesterol levels by promoting RCT. SIRT1 is known to play a beneficial role also in RCT, which is through the regulation of hepatic expression of the two primary HDL receptors, ABCA1 and SR-B1 [7,9]. Several studies demonstrated that SIRT1 not only activates LXRα to upregulate ABCA1, a key player in nascent HDL biogenesis, but also stimulates PGC-1α-dependent expression of SR-B1. Therefore, together with our data showing an upregulated expression of SIRT1 and RCT-related genes in the liver of *L*. *plantarum* treated mice (Fig 2H and 2L), it is suggested that the *L*. *plantarum* treatment-mediated improvement of RCT is attributed to, at least in part, an activation of SIRT1/PGC-1α pathway. Although a large number of studies have shown that supplementation with probiotic bacteria such as *Lactobacillus* and *Bifidobacterium* improves serum lipid profiles, including reduced total cholesterol and TG, as well as increased HDL-cholesterol, in animals and humans [9, 39, 40], there have been no clear elucidation of the mechanisms driving these improvements. Our findings in this study provide additional evidence for hypocholesterolemic potential of *Lactobacillus* strains and also new insights into mechanism by which *Lactobacillus* probiotics ameliorate the cholesterol metabolic dysfunctions. In the mechanism, SIRT1 is particularly highlighted for its potential role in lowering serum total cholesterol while elevating HDL-cholesterol levels.

Accumulated evidence show that, under metabolic disorder conditions, intracellular NAD^+^ levels decreased in metabolic tissues, including the liver, adipose tissue, skeletal muscle, which is associated with decreased activation of SIRT1, a NAD+-dependent deacetylase [41,42]. It has been demonstrated that adiponectin reverses this metabolic state by increasing cellular NAD^+^ levels via activation of AMPK, leading to the activation of SIRT1/PGC-1α pathway [11, 26]. Thus, the activation of adiponectin-AMPK-SIRT1 pathway could be one potential mechanism underlying the protective effect of adiponectin against obesity-related disorders. Data obtained in this study show that, besides the upregulated expression of SIRT1, *L*. *plantarum* treatment also augmented the production of adiponectin in adipose tissues including MAT, SAT, and BAT (Fig 2I), which might increase the activity of SIRT1, leading to further potentiation of the protective effect of SIRT1/PGC-1α against metabolic dysfunctions. This explanation is supported by the observations that, accompanied by an increase in adiponectin production, the expression of adiponectin receptors, AdipoR1 and AdipoR2, were also significantly upregulated in the liver and adipose tissue of *L*. *plantarum* treated mice (Fig 2J). Collectively, these results suggest that *L*. *plantarum*-induced beneficial effects on bile acid and cholesterol metabolism may be attributed to not only elevated expression of SIRT1, but also promoted SIRT1 activity resulting from enhanced adiponectin production.

In efforts to develop new approaches in the fight against metabolic disorders, the discovery of metabolic hormones such as irisin and FGF21 and their potential to induce the browning of WAT, especially SAT, and the thermogenic capacity of BAT has gained great interest. Irisin, a PGC-1α-dependent thermogenic adipomyokine produced by skeletal muscle and adipose tissue, is known to have multiple functions, such as enhancing insulin sensitivity in skeletal muscle, improving hepatic glucose and lipid metabolism, and promoting WAT browning [43]. In particular, irisin has been shown to promote thermogenesis in BAT by upregulating UCP1 expression and also stimulate browning by increasing the expression BAT-specific genes and UCP1 in SAT [15,44]. FGF21 also acts as a key regulator in SAT browning and BAT thermogenesis, which is primarily by inducing PGC1α-mediated mitochondrial biogenesis and UCP1 expression [18]. Apart from irisin and FGF21, bile acids also promote WAT browning (TGR5-dependent induction of WAT-resident brown-like adipocytes) and BAT thermogenic energy expenditure (via controlling TGR5-cAMP-Dio2 signaling) [45] by upregulating mitochondrial function-related genes, which is mediated by PGC-1α activation [33]. SIRT1 is also known to induce WAT browning by deacetylating PPARγ, leading to its recruitment to Prdm16 and PGC-1α and transcription of BAT-specific genes [46], and enhance BAT thermogenesis by potentiating PPARα/PGC-1α-mediated mitochondrial functions [47]. Our results showed that *L*. *plantarum* treatment increased the expression of genes involved in WAT browning and BAT thermogenesis, including ND5, Prdm16, Cidea, Elov13, and UCP1 (Fig 3D and 3E), and their master regulators PPARα and PGC-1α (Fig 4G and 4I). These findings, taken together with the data showing that expressions of irisin, FGF21, bile acid synthetic genes, TGR5, and SIRT1 are upregulated in SAT and BAT (Figs 2B–2G, 2L, 3A and 3B), suggest that the protective effect of *L*. *plantarum* treatment against HFD-induced dyslipidemia is associated with enhanced irisin-, FGF21-, and bile acid-mediated WAT browning and BAT thermogenesis.

We further hypothesized that the increased SIRT1 expression in the liver, MAT, SAT, and BAT of *L*. *plantarum* treated mice would lead to the reversal of HFD-induced fat deposition in the tissues. It has been demonstrated that SIRT1 promotes lipid oxidation through PPARα/PGC-1α pathway, leading to reduction of fat accumulation [8]. As expected from the data showing a *L*. *plantarum* treatment-mediated enhancement of SIRT1 expression (Fig 2L), we observed that, along with histologically observed reductions in hepatic steatosis (as well as hepatic TG content) and adipocyte size of adipose tissues, the expression of lipid oxidation-related genes was upregulated by *L*. *plantarum* treatment whereas the expression levels of lipid synthetic genes remained unaltered (Figs 4 and S1). These results demonstrate that the treatment with *L*. *plantarum* ameliorates both HFD-induced hepatic fat deposition and adipose tissue fat storage by increasing lipid oxidation, but not suppressing lipid synthesis, which is mediated through SIRT1-PGC-1α pathway.

It is interesting that all the effects exerted by *L*. *plantarum* treatment, including enhancement of bile acid synthesis and RCT in the liver, improvement of WAT browning and BAT thermogenesis, and attenuation of fat accumulation in the liver and adipose tissue, are commonly mediated by activation of PGC-1α. Most of these effects are via SIRT1/PGC-1α-mediated mitochondrial regulation. Considering that the inter-organ crosstalk is critical in metabolic homeostasis, the discovery of a concurrent mechanism underlying the metabolically beneficial effects is challenging because a single probiotics agent usually exerts multiple effects simultaneously in different tissues. In this study, the PGC-1α-mediated pathway was discovered as a concurrent mechanism that may account for the comprehensive improvement effect of *L*. *plantarum* treatment on metabolic disorders.

It is important to understand the role of the gut microbiota dysbiosis in the pathogenesis of metabolic disorders for developing approaches to therapeutic modulation of microbiota by use of probiotics. It has been known that the most predominant phyla are Firmicutes and Bacteroidetes in both humans and mice, and the others include Actinobacteria, Proteobacteria, Deferribacteres, and Verrucomicrobia [48]. Studies have shown that an increased Firmicutes/Bacteroidetes (F/B) ratio is involved in the development of obesity in mice and humans [49]. However, some studies have found controversial results, including a report demonstrating no difference in the proportions of Bacteroidetes and Firmicutes between lean and obese subjects [50]. There is now substantial amount of evidence to indicate that HFD-induced obesity is associated with alterations in gut microbiota composition, including a decrease in Bacteroidetes and increase in Firmicutes abundance in both mice and humans [51–53]. Consistent with previous studies, we observed in this study that HFD-fed mice had decreased abundance of Bacteroidetes, but unexpectedly, unaltered proportion of Firmicutes (Fig 5C). An extensive metagenomics study has discovered a lower Bacteroidetes, but, interestingly, and a higher Actinobacteria abundance in obese than lean subjects, while no significant difference in Firmicutes proportion [54]. Also similar to humans, HFD-fed mice have been shown to have an increased abundance of Actinobacteria [55, 56]. This is similar to our results showing a decreased Bacteroidetes and increased Actinobacteria abundance in HFD-fed mice while Firmicutes proportion remains unchanged (Fig 5C). We also observed that the HFD-induced decrease in Bacteroidetes and increase in Actinobacteria were reversed by *L*. *plantarum* treatment (Fig 5C). Additionally, at the class level, *L*. *plantarum* treatment reduced the ratio of *Clostridia* to *Bacteroidia*, which are Firmicutes- and Bacteroidetes-associated class, respectively. This is also consistent with several studies reporting a reduced abundance of *Bacteroidia* or an increased *Clostridia*/*Bacteroidia* ratio in HFD-fed animals [57,58]. Further, at the family level, we also found that *Rikenellaceae*, *Ruminococcaceae*, and *Lachnospiraceae* families enriched by HFD feeding were decreased by *L*. *plantarum* treatment (Fig 5D). The abundances of these families have already been reported to be positively correlated with HFD-induced obesity, for example, enriched proportions of *Rikenellaceae* and *Ruminococcaceae* in obese-diabetes model (db/db) mice [59] and HFD-fed mice [60], and of *Ruminococcaceae* and *Lachnospiraceae* in HFD-fed mice [61,62].

Recent studies have also shown that bile acids appear to play a critical role in regulating gut microbiota [63]. Reported results include the decreased abundance of *Lachnospiraceae* in HFD-fed mice following bile acid supplementation [64], the negative correlations of *Ruminococcaceae* abundance with CYP7A1 (the rate-limiting enzyme for bile acid synthesis) expression and *Lachnospiraceae* with adenlyate cyclase 7 (Adcy7, downstream target of bile acid receptor) expression [65], and the positive correlation of abundance of *Lachnospirace* and *Ruminococcaceae* with the levels of plasma total and LDL cholesterol [66]. Therefore, the results of gut microbiota analysis in this study showing reduced abundance of phylum Actinobacteria, families *Rikenellaceae*, *Ruminococcaceae*, and *Lachnospiraceae*, and lowered ratio of *Clostridia/Bacteroidia* class in *L*. *plantarum* treated mice, demonstrate that the *L*. *plantarum* treatment-medicated attenuation of metabolic dysfunction is associated with probiotic-mediated modulation of HFD-induced dysbiosis.

**Fig 6:**
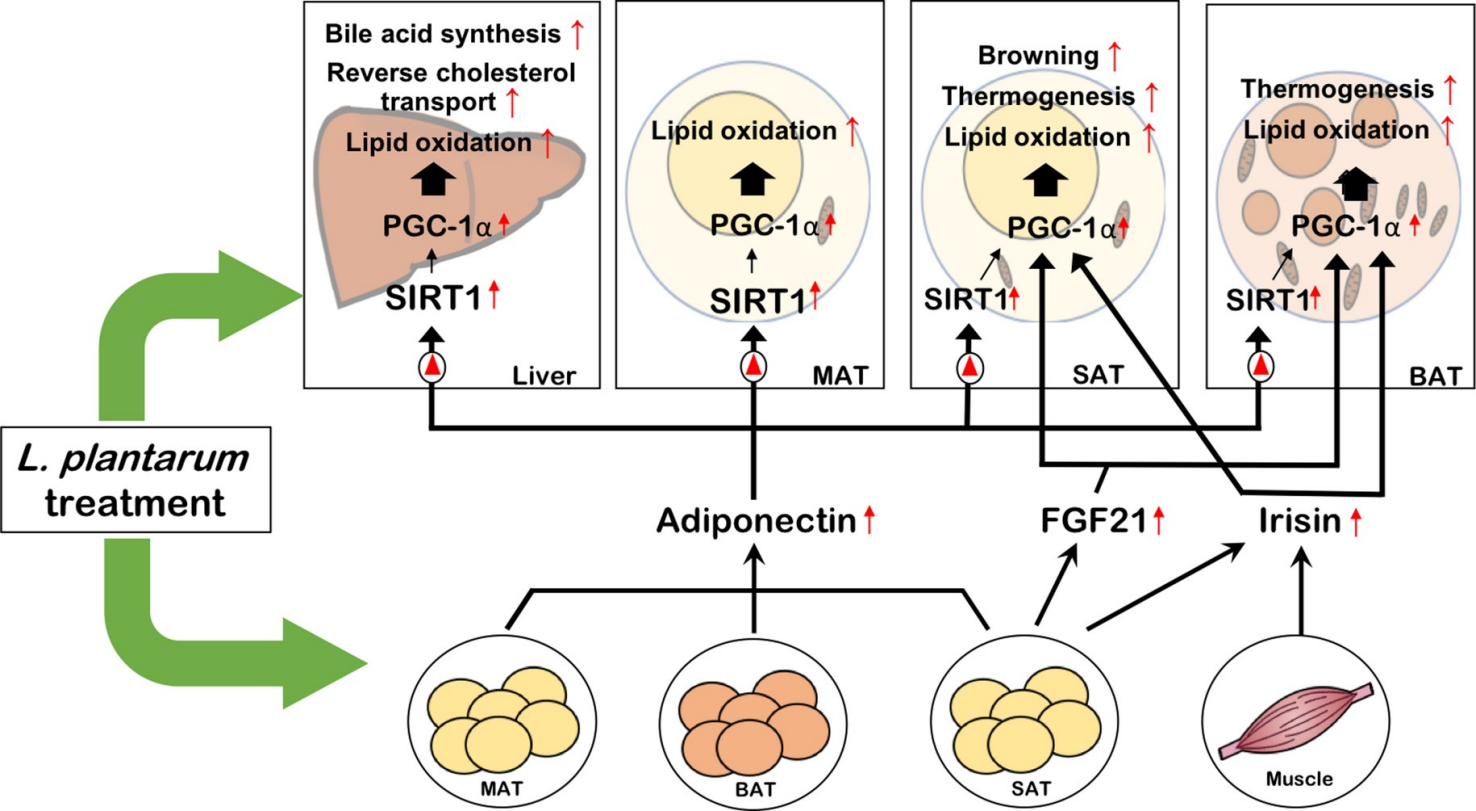
A summary on the protective effects of *L*. *plantarum* probiotics against high-fat diet-induced metabolic dysfunctions through activation of the PGC-1α pathway.

In summary, the *L*. *plantarum* strain used in this study exerted a comprehensive ameliorating effect on metabolic disorders, including adiposity, glucose intolerance and dyslipidemia. These *L*. *plantarum* treatment-mediated improvements were associated with improved bile acid and cholesterol homeostasis, enhanced adipose tissue browning and thermogenesis, and reduced fat accumulation, all of which are commonly mediated by activation of PGC-1α. All these metabolic benefits of *L*. *plantarum* treatment also might be associated with probiotic reversal of HFD-induced gut microbiota dysbiosis. Taken our findings together, here we propose a model that describes the probiotic effect of *L*. *plantarum* ameliorating metabolic disorders as shown in Fig 6. It shows that the treatment of *L*. *plantarum* stimulates the expression of SIRT1, PPARα, and PGC-1α in the liver and adipose tissues, leading to increased bile acid synthesis and RCT in the liver, enhanced WAT browning and BAT thermogenesis, and increased fatty acid oxidation in the liver and adipose tissue, which are mediated by upregulated adiponectin, irisin, and/or FGF21 via SIRT1-dependent or independent activation of PGC-1α pathway. Based on these findings, the PGC-1α-mediated pathway could represent a potential target for molecular therapy in the probiotics-based approach to ameliorate metabolic disorders.

## Supporting information

S1 Fig There is no difference in lipogenic gene expression in the liver and adipose tissue. (A-D) Lipogenic gene expression in the liver, MAT, SAT, and BAT, respectively. All genes are normalized to expression of β-actin. Data present mean ± SD for 7~8 mice in each group. Student’s two-tailed t-test was used for analysis difference between experimental groups. Student’s two-tailed t-test was used for analysis of differences between groups. ^#, •, +, *^p < 0.05, ^##, ••, ++, **^ *p* < 0.01, ^###, •••, +++, ***^ *p* < 0.001.
(TIF)

S2 Fig Raw images of Fig 2K. (PDF)

S1 Table Composition of the normal diet and high-fat diet. (DOCX)

S2 Table Primer sequences for real-time PCR. (DOCX)

## References

[1] Canfora EE, Meex RCR, Venema K, Blaak EE. Gut microbial metabolites in obesity, NAFLD and T2DM. Nat Rev Endocrinol. 2019; 15(5): 261–273. 10.1038/s41574-019-0156-z 30670819

[2] Paolella G, Mandato C, Pierri L, Poeta M, Di Stasi M, Vajro P. Gut-liver axis and probiotics: their role in non-alcoholic fatty liver disease. World J Gastroenterol. 2014; 20(42): 15518–15531. 10.3748/wjg.v20.i42.15518 25400436 PMC4229517

[3] He M, Shi B. Gut microbiota as a potential target of metabolic syndrome: the role of probiotics and prebiotics. Cell Biosci. 2017; 7: 54 10.1186/s13578-017-0183-1 29090088 PMC5655955

[4] Zhao X, Higashikawa F, Noda M, Kawamura Y, Matoba Y, Kumagai T, et al The obesity and fatty liver are reduced by plant-derived Pediococcus pentosaceus LP28 in high fat diet-induced obese mice. PLoS One. 2012; 7(2): e30696 10.1371/journal.pone.0030696 22363472 PMC3281851

[5] Bagarolli RA, Tobar N, Oliveira AG, Araújo TG, Carvalho BM, Rocha GZ, et al Probiotics modulate gut microbiota and improve insulin sensitivity in DIO mice. J Nutr Biochem. 2017; 50: 16–25. 10.1016/j.jnutbio.2017.08.006 28968517

[6] Ritze Y, Bárdos G, Claus A, Ehrmann V, Bergheim I, Schwiertz A, et al Lactobacillus rhamnosus GG protects against non-alcoholic fatty liver disease in mice. PLoS One. 2014; 9(1): e80169 10.1371/journal.pone.0080169 24475018 PMC3903470

[7] Chang HC, Guarente L. SIRT1 and other sirtuins in metabolism. Trends Endocrinol Metab. 2014; 25(3): 138–145. 10.1016/j.tem.2013.12.001 24388149 PMC3943707

[8] Purushotham A, Schug TT, Xu Q, Surapureddi S, Guo X, Li X. Hepatocyte-specific deletion of SIRT1 alters fatty acid metabolism and results in hepatic steatosis and inflammation. Cell Metab. 2009; 9(4): 327–338. 10.1016/j.cmet.2009.02.006 19356714 PMC2668535

[9] Rodgers JT, Puigserver P. Fasting-dependent glucose and lipid metabolic response through hepatic sirtuin 1. Proc Natl Acad Sci U S A. 2007; 104(31): 12861–12866. 10.1073/pnas.0702509104 17646659 PMC1937557

[10] Ruan H, Dong LQ. Adiponectin signaling and function in insulin target tissues. J Mol Cell Biol. 2016; 8(2): 101–109. 10.1093/jmcb/mjw014 26993044 PMC4816150

[11] Iwabu M, Yamauchi T, Okada-Iwabu M, Sato K, Nakagawa T, Funata M, et al Adiponectin and AdipoR1 regulate PGC-1alpha and mitochondria by Ca(2+) and AMPK/SIRT1. Nature. 2010; 464(7293): 1313–1319. 10.1038/nature08991 20357764

[12] Fillmore N, Jacobs DL, Mills DB, Winder WW, Hancock CR. Chronic AMP-activated protein kinase activation and a high-fat diet have an additive effect on mitochondria in rat skeletal muscle. J Appl Physiol (1985). 2010; 109(2): 511–520. 20522731 10.1152/japplphysiol.00126.2010 PMC2928588

[13] Achari AE, Jain SK. Adiponectin, a therapeutic target for obesity, diabetes, and endothelial dysfunction. Int J Mol Sci. 2017; 18(6). pii: E1321 10.3390/ijms18061321 28635626 PMC5486142

[14] Lee P, Linderman JD, Smith S, Brychta RJ, Wang J, Idelson C, et al Irisin and FGF21 are cold-induced endocrine activators of brown fat function in humans. Cell Metab. 2014; 19(2): 302–309. 10.1016/j.cmet.2013.12.017 24506871 PMC7647184

[15] Boström P, Wu J, Jedrychowski MP, Korde A, Ye L, Lo JC, Rasbach KA, et al A PGC1-α-dependent myokine that drives brown-fat-like development of white fat and thermogenesis. Nature. 2012; 481(7382): 463–468. 10.1038/nature10777 22237023 PMC3522098

[16] Zhang Y, Xie C, Wang H, Foss RM, Clare M, George EV, et al Irisin exerts dual effects on browning and adipogenesis of human white adipocytes. Am J Physiol Endocrinol Metab. 2016; 311(2): E530–E541. 10.1152/ajpendo.00094.2016 27436609

[17] Fisher FM, Kleiner S, Douris N, Fox EC, Mepani RJ, Verdeguer F, et al FGF21 regulates PGC-1α and browning of white adipose tissues in adaptive thermogenesis. Genes Dev. 2012; 26(3): 271–281. 10.1101/gad.177857.111 22302939 PMC3278894

[18] Cuevas-Ramos D, Mehta R, Aguilar-Salinas CA. Fibroblast growth factor 21 and browning of white adipose tissue. Front Physiol. 2019; 10: 37 10.3389/fphys.2019.00037 30804796 PMC6370737

[19] Marlatt KL, Ravussin E. Brown adipose tissue: an update on recent findings. Curr Obes Rep. 2017; 6(4): 389–396. 10.1007/s13679-017-0283-6 29101739 PMC5777285

[20] Reynés B, Palou M, Rodríguez AM, Palou A. Regulation of adaptive thermogenesis and browning by prebiotics and postbiotics. Front Physiol. 2019; 9: 1908 10.3389/fphys.2018.01908 30687123 PMC6335971

[21] Lim SD, Kim KS, Do JR. Physiological characteristics and production of vitamin K2 by Lactobacillus fermentum LC272 isolated from raw milk. Food Sci. Anim. Resour. 2011; 31: 513–520.

[22] Kim B, Kwon J, Kim MS, Park H, Ji Y, Holzapfel W, Hyun CK. Protective effects of Bacillus probiotics against high-fat diet-induced metabolic disorders in mice. PLoS One. 2018; 13(12): e0210120 10.1371/journal.pone.0210120 30596786 PMC6312313

[23] Galarraga M, Campión J, Muñoz-Barrutia A, Boqué N, Moreno H, Martínez JA, et al Adiposoft: automated software for the analysis of white adipose tissue cellularity in histological sections. J Lipid Res. 2012; 53(12): 2791–2796. 10.1194/jlr.D023788 22993232 PMC3494244

[24] Amplicon PCR, clean‐up PCR, index PCR. 16s metagenomic sequencing library preparation. 2013.

[25] You M, Rogers CQ. Adiponectin: a key adipokine in alcoholic fatty liver. Exp Biol Med (Maywood). 2009; 234(8): 850–859. 19491377 10.3181/0902-MR-61

[26] Shen Z, Liang S, Rogers CQ, Rideout D, You M. Involvement of adiponectin-SIRT1-AMPK signaling in the protective action of rosiglitazone against alcoholic fatty liver in mice. Am J Physiol Gastrointest Liver Physiol. 2010; 298(3): G364–G374. 10.1152/ajpgi.00456.2009 20007851 PMC2838513

[27] Velazquez-Villegas LA, Perino A, Lemos V, Zietak M, Nomura M, Pols TWH, et al TGR5 signalling promotes mitochondrial fission and beige remodelling of white adipose tissue. Nat Commun. 2018; 9(1): 245 10.1038/s41467-017-02068-0 29339725 PMC5770450

[28] Broeders EP, Nascimento EB, Havekes B, Brans B, Roumans KH, Tailleux A, et al The bile acid chenodeoxycholic acid increases human brown adipose tissue activity. Cell Metab. 2015; 22(3): 418–426. 10.1016/j.cmet.2015.07.002 26235421

[29] Schug TT, Li X. Sirtuin 1 in lipid metabolism and obesity. Ann Med. 2011; 43(3): 198–211. 10.3109/07853890.2010.547211 21345154 PMC3173813

[30] Elibol B, Kilic U. High levels of SIRT1 expression as a protective mechanism against disease-related conditions. Front Endocrinol (Lausanne). 2018; 9: 614. 30374331 10.3389/fendo.2018.00614 PMC6196295

[31] Ðanić M, Stanimirov B, Pavlović N, Goločorbin-Kon S, Al-Salami H, Stankov K, et al Pharmacological applications of bile acids and their derivatives in the treatment of metabolic syndrome. Front Pharmacol. 2018; 9: 1382 10.3389/fphar.2018.01382 30559664 PMC6287190

[32] Flynn CR, Albaugh VL, Cai S, Cheung-Flynn J, Williams PE, Brucker RM, et al Bile diversion to the distal small intestine has comparable metabolic benefits to bariatric surgery. Nat Commun. 2015; 6: 7715 10.1038/ncomms8715 26197299 PMC4518285

[33] Pierre JF, Martinez KB, Ye H, Nadimpalli A, Morton TC, Yang J, et al Activation of bile acid signaling improves metabolic phenotypes in high-fat diet-induced obese mice. Am J Physiol Gastrointest Liver Physiol. 2016; 311(2): G286–G304. 10.1152/ajpgi.00202.2016 27340128 PMC5007288

[34] Chiang JY. Bile acid metabolism and signaling. Compr Physiol. 2013; 3(3): 1191–1212. 10.1002/cphy.c120023 23897684 PMC4422175

[35] Schapp FG, Trauner M, Jansen PL. Bile acid receptors as targets for drug development. Nat Rev Gastroenterol Hepatol. 2014; 11(1): 55–67. 10.1038/nrgastro.2013.151 23982684

[36] Owen BM, Mangelsdorf DJ, Kliewer SA. Tissue-specific actions of the metabolic hormones FGF15/19 and FGF21. Trends Endocrinol Metab. 2015; 26(1): 22–29. 10.1016/j.tem.2014.10.002 25476453 PMC4277911

[37] Afonso MS, Machado RM, Lavrador MS, Quintao ECR, 4 Moore KJ, Lottenberg AM. Molecular pathways underlying cholesterol homeostasis. Nutrients. 2018; 10(6). pii: E760 10.3390/nu10060760 29899250 PMC6024674

[38] Marques LR, Diniz TA, Antunes BM, Rossi FE, Caperuto EC, Lira FS. Reverse cholesterol transport: molecular mechanisms and the non-medical approach to enhance HDL cholesterol. Front Physiol. 2018; 9: 526 10.3389/fphys.2018.00526 29867567 PMC5962737

[39] Thushara RM, Gangadaran S, Solati Z, Moghadasian MH. Cardiovascular benefits of probiotics: a review of experimental and clinical studies. Food Funct. 2016; 7(2): 632–642. 10.1039/c5fo01190f 26786971

[40] Wu Y, Zhang Q, Ren Y, Ruan Z. Effect of probiotic Lactobacillus on lipid profile: a systematic review and meta-analysis of randomized, controlled trials. PloS one. 2017; 12(6): e0178868 10.1371/journal.pone.0178868 28594860 PMC5464580

[41] Cantó C, Houtkooper RH, Pirinen E, Youn DY, Oosterveer MH, Cen Y. The NAD(+) precursor nicotinamide riboside enhances oxidative metabolism and protects against high-fat diet-induced obesity. Cantó et al., Cell Metab. 2012; 15(6): 838–847. 10.1016/j.cmet.2012.04.022 22682224 PMC3616313

[42] Kraus D, Yang Q, Kong D, Banks AS, Zhang L, Rodgers JT. Nicotinamide N-methyltransferase knockdown protects against diet-induced obesity. Nature. 2014; 508(7495): 258–262. 10.1038/nature13198 24717514 PMC4107212

[43] Arhire LI, Mihalache L, Covasa M. Irisin: A hope in understanding and managing obesity and metabolic syndrome. Front Endocrinol (Lausanne). 2019; 10: 524. 31428053 10.3389/fendo.2019.00524 PMC6687775

[44] Zhang Y, Li R, Meng Y, Li S, Donelan W, Zhao Y, et al Irisin stimulates browning of white adipocytes through mitogen-activated protein kinase p38 MAP kinase and ERK MAP kinase signaling. Diabetes. 2014; 63(2): 514–525. 10.2337/db13-1106 24150604 PMC13117908

[45] Watanabe M, 1 Houten SM, Mataki C, Christoffolete MA, Kim BW, Sato H, et al Bile acids induce energy expenditure by promoting intracellular thyroid hormone activation. Nature. 2006; 439(7075): 484–489. 10.1038/nature04330 16400329

[46] Qiang L, Wang L, Kon N, Zhao W, Lee S, Zhang Y, et al Brown remodeling of white adipose tissue by Sirt1-dependent deacetylation of PPARγ. Cell. 2012; 150(3): 620–632. 10.1016/j.cell.2012.06.027 22863012 PMC3413172

[47] Boutant M, Joffraud M, Kulkarni SS, García-Casarrubios E, García-Roves PM, Ratajczak J, et al SIRT1 enhances glucose tolerance by potentiating brown adipose tissue function. Mol Metab. 2014; 4(2): 118–131. 10.1016/j.molmet.2014.12.008 25685699 PMC4314542

[48] Rinninella Emanuele, Raoul Pauline, Cintoni Marco, Franceschi Francesco, Miggiano Giacinto Abele Donato, Gasbarrini Antonio, et al What is the healthy gut microbiota composition? A changing ecosystem across age, environment, diet, and diseases. Microorganisms. 2019; 7(1): 14. 10.3390/microorganisms7010014 PMC6351938 30634578

[49] Dalby MJ, Ross AW, Walker AW, Morgan PJ. Dietary uncoupling of gut microbiota and energy harvesting from obesity and glucose tolerance in mice. Cell Rep. 2017; 21(6): 1521–1533. 10.1016/j.celrep.2017.10.056 29117558 PMC5695904

[50] Duncan SH, Lobley GE, Holtrop G, Ince J, Johnstone AM, Louis P, Flint HJ. Human colonic microbiota associated with diet, obesity and weight loss. Int J Obes (Lond). 2008; 32(11): 1720–1724. 18779823 10.1038/ijo.2008.155

[51] Turnbaugh PJ, Bäckhed F, Fulton L, Gordon JI. Diet-induced obesity is linked to marked but reversible alterations in the mouse distal gut microbiome. Cell Host Microbe. 2008; 3: 213–223. 10.1016/j.chom.2008.02.015 18407065 PMC3687783

[52] Delzenne NM, Neyrinck AM, Bäckhed F, Cani PD. Targeting gut microbiota in obesity: effects of prebiotics and probiotics. Nat Rev Endocrinol. 2011; 7(11): 639–646. 10.1038/nrendo.2011.126 21826100

[53] David LA, Maurice CF, Carmody RN, Gootenberg DB, Button JE, Wolfe BE, et al Diet rapidly and reproducibly alters the human gut microbiome. Nature. 2014; 505(7484): 559–563. 10.1038/nature12820 24336217 PMC3957428

[54] Turnbaugh PJ, 1 Hamady M, Yatsunenko T, Cantarel BL, Duncan A, Ley RE, et al A core gut microbiome in obese and lean twins. Nature. 2009; 457(7228): 480–484. 10.1038/nature07540 19043404 PMC2677729

[55] Hildebrandt MA, Hoffmann C, Sherrill-Mix SA, Keilbaugh SA, Hamady M, Chen YY, et al High fat diet determines the composition of the murine gut microbiome independently of obesity. Gastroenterology. 2009; 137(5): 1716–1724. e1-2. 10.1053/j.gastro.2009.08.042 PMC2770164 19706296

[56] Clarke SF, Murphy EF, Nilaweera K, Ross PR, Shanahan F, O'Toole PW, et al The gut microbiota and its relationship to diet and obesity: new insights. Gut Microbes. 2012; 3(3): 186–202. 10.4161/gmic.20168 22572830 PMC3427212

[57] Goodman AL, Kallstrom G, Faith JJ, Reyes A, Moore A, Dantas G, et al Extensive personal human gut microbiota culture collections characterized and manipulated in gnotobiotic mice. Proc Natl Acad Sci U S A. 2011; 108(15): 6252–6257. 10.1073/pnas.1102938108 21436049 PMC3076821

[58] Lee SM. Kim N, Yoon H, Nam RH, Lee DH. Microbial changes and host response in F344 rat colon depending on sex and age following a high-fat diet. Front Microbiol. 2018; 9: 2236 10.3389/fmicb.2018.02236 30298061 PMC6160749

[59] Geurts L, 1 Lazarevic V, Derrien M, Everard A, Van Roye M, Knauf C, et al Altered gut microbiota and endocannabinoid system tone in obese and diabetic leptin-resistant mice: impact on apelin regulation in adipose tissue. Front Microbiol. 2011; 2: 149 10.3389/fmicb.2011.00149 21808634 PMC3139240

[60] Kim KA, Gu W, Lee IA, Joh EH, Kim DH, et al High fat diet-induced gut microbiota exacerbates inflammation and obesity in mice via the TLR4 signaling pathway. PLoS one. 2012; 7(10): e47713 10.1371/journal.pone.0047713 23091640 PMC3473013

[61] Evans CC, LePard KJ, Kwak JW, Stancukas MC, Laskowski S, Dougherty J, et al Exercise prevents weight gain and alters the gut microbiota in a mouse model of high fat diet-induced obesity. PLoS One. 2014; 9(3): e92193 10.1371/journal.pone.0092193 24670791 PMC3966766

[62] Zeng H, Ishaq SL, Zhao FQ, Wright AG. Colonic inflammation accompanies an increase of β-catenin signaling and Lachnospiraceae/Streptococcaceae bacteria in the hind gut of high-fat diet-fed mice. J Nutr Biochem. 2016; 35: 30–36. 10.1016/j.jnutbio.2016.05.015 27362974

[63] Ridlon JM, Kang DJ, Hylemon PB, Bajaj JS. Bile acids and the gut microbiome. Curr Opin Gastroenterol. 2014; 30(3): 332–338. 10.1097/MOG.0000000000000057 24625896 PMC4215539

[64] Just S, Mondot S, Ecker J, Wegner K, Rath E, Gau L, et al The gut microbiota drives the impact of bile acids and fat source in diet on mouse metabolism. Microbiome. 2018; 6(1): 134 10.1186/s40168-018-0510-8 30071904 PMC6091023

[65] Liu HX, Rocha CS, Dandekar S, Wan YJ. Functional analysis of the relationship between intestinal microbiota and the expression of hepatic genes and pathways during the course of liver regeneration. J Hepatol. 2016; 64(3): 641–650. 10.1016/j.jhep.2015.09.022 26453969 PMC4761311

[66] Liu S, Bennett DC, Tun HM, Kim JE, Cheng KM, Zhang H, et al The effect of diet and host genotype on ceca microbiota of Japanese quail fed a cholesterol enriched diet. Front Microbiol. 2015; 6: 1092 10.3389/fmicb.2015.01092 26500632 PMC4595795

