# Supplementary figures and images for: Comprehensive amelioration of high-fat diet-induced metabolic dysfunctions through activation of the PGC-1α pathway by probiotics treatment in mice
Source: PLoS One. 2020 Feb 10;15(2):e0228932. doi: 10.1371/journal.pone.0228932 (PMC7010303; doi:10.1371/journal.pone.0228932)

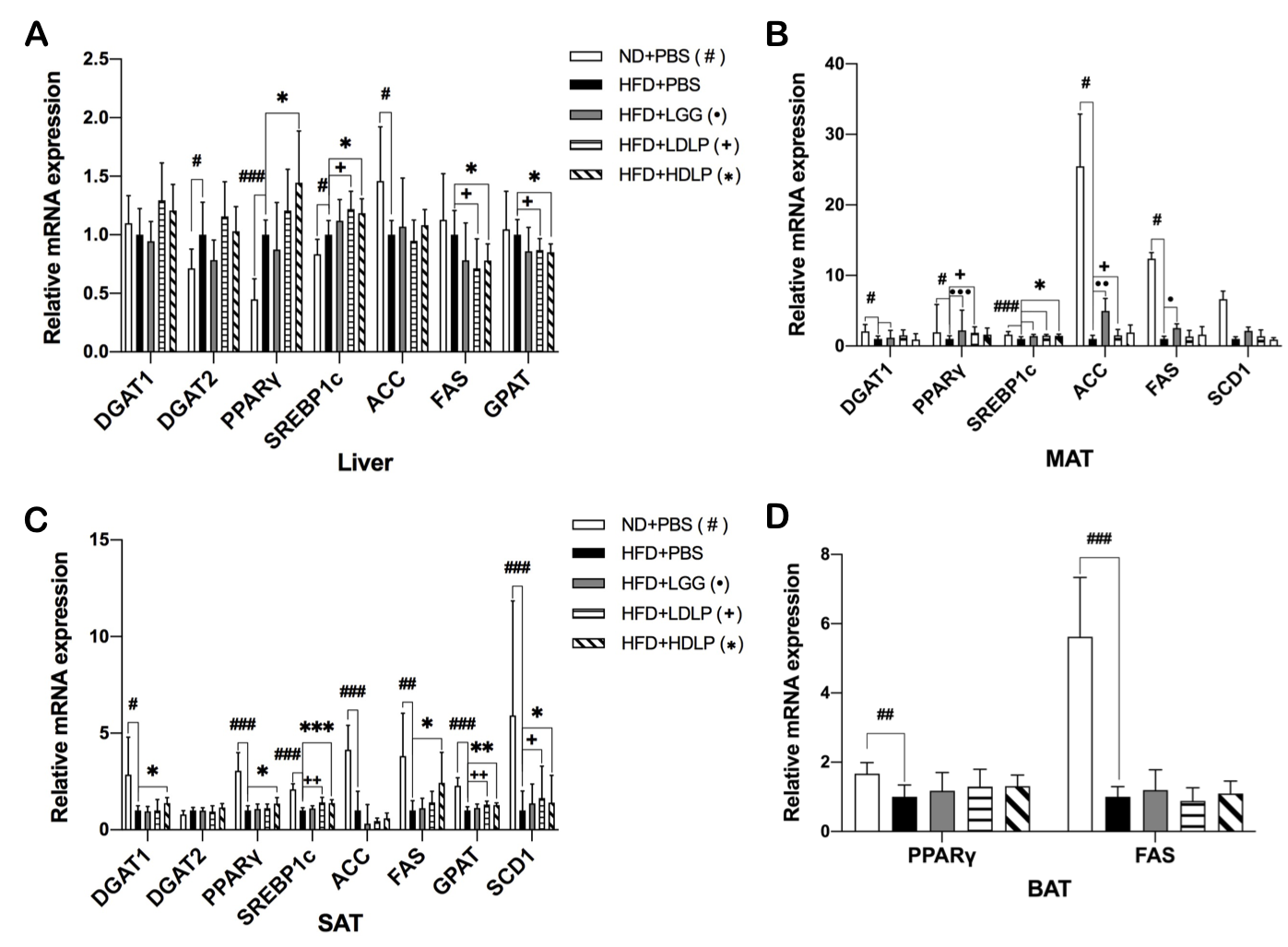

Supplement: S1 Fig — (A-D) Lipogenic gene expression in the liver, MAT, SAT, and BAT, respectively. All genes are normalized to expression of β-actin. Data present mean ± SD for 7~8 mice in each group. Student’s two-tailed t-test was used for analysis difference between experimental groups. Student’s two-tailed t-test was used for analysis of differences between groups. #, •, +, *p < 0.05, ##, ••, ++, ** p < 0.01, ###, •••, +++, *** p < 0.001. (TIF) [file pone.0228932.s001.tif]
