# Supplementary material for: Comprehensive amelioration of high-fat diet-induced metabolic dysfunctions through activation of the PGC-1α pathway by probiotics treatment in mice
Source: PLoS One. 2020 Feb 10;15(2):e0228932. doi: 10.1371/journal.pone.0228932 (PMC7010303; doi:10.1371/journal.pone.0228932)
Supplement: S2 Fig — (PDF) [file pone.0228932.s002.pdf]

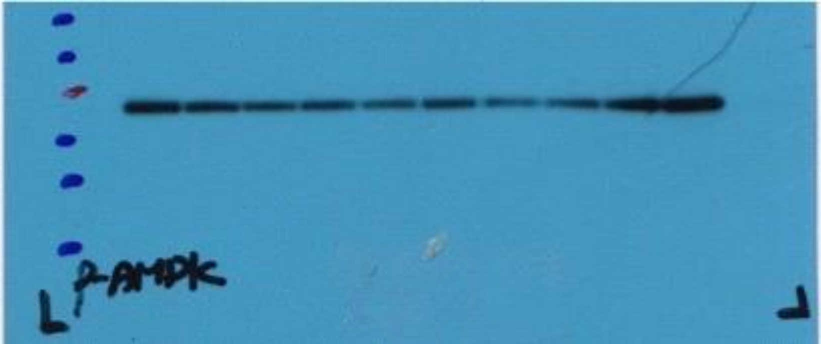
P-AMPK

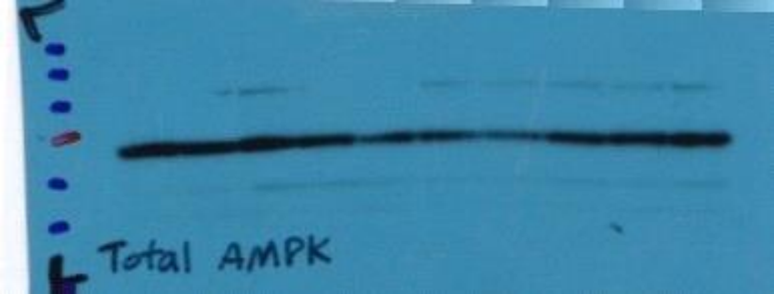
Total AMPK
