# Supplementary material for: Comprehensive amelioration of high-fat diet-induced metabolic dysfunctions through activation of the PGC-1α pathway by probiotics treatment in mice
Source: PLoS One. 2020 Feb 10;15(2):e0228932. doi: 10.1371/journal.pone.0228932 (PMC7010303; doi:10.1371/journal.pone.0228932)
Supplement: S1 Table — (DOCX) [file pone.0228932.s003.docx]

**Supporting Table 1. Composition of Diet**

| **Diet (% w/w)** | **Normal Diet** | **High-fat diet** |
| --- | --- | --- |
| Protein | 66.7 | 26.2 |
| Carbohydrate | 19.2 | 25.6 |
| Fiber | 4.7 | 6.5 |
| Fat | 4.3 | 34.9 |
| Vitamin mix | 0.3 | 0.3 |
| Mineral mix | 4.7 | 6.5 |
| Energy density (kcal/g) | 3.82 | 5.21 |

* Diets were not sterilized.
