# Supplementary material for: Comprehensive amelioration of high-fat diet-induced metabolic dysfunctions through activation of the PGC-1α pathway by probiotics treatment in mice
Source: PLoS One. 2020 Feb 10;15(2):e0228932. doi: 10.1371/journal.pone.0228932 (PMC7010303; doi:10.1371/journal.pone.0228932)
Supplement: S2 Table — (DOCX) [file pone.0228932.s004.docx]

**Supporting Table 2. Primer sequences for real-time PCR**

| **Gene** | | **Sequence** |
| --- | --- | --- |
| CYP7A1 | Forward | GGTCCTCCAGCAGAGAGCTA |
|  | Reverse | AGGAAGGAAGCATAGCGTACC |
| CYP7B1 | Forward | TAGCCCTCTTTCCTCCACTCATA |
|  | Reverse | GAACCGATCGAACCTAAATTCCT |
| CYP27A1 | Forward | AGTGATGAGACAGGAGGGCA |
|  | Reverse | TCCTTGTGCGATGAAGATCCC |
| CYP8B1 | Forward | CTGGGTCACTCCATGGCTTT |
|  | Reverse | GGAAGTCCAGCGCTTTCTCT |
| BSEP | Forward | CTGCCAAGGATGCTAATGCA |
|  | Reverse | CGATGGCTACCCTTTGCTTCT |
| β-klotho | Forward | GGTCTCCGGGGAATGAATGG |
|  | Reverse | TAACAGCTCGCAGCAGAACA |
| FGFR1C | Forward | GTAGCTCCCTACTGGACATCC |
|  | Reverse | GCATAGCGAACCTTGTAGCCTC |
| FGFG4 | Forward | AGGTGGTCAGTGGGAAGTCTG |
|  | Reverse | CTGCTCCAGGATTGGGGCTA |
| TGR5 | Forward | CTT CTC TCT GTC CGC GTG TT |
|  | Reverse | GCC AGG GTT GAG GGT ACA TC |
| FGF15 | Forward | GACTGCGAGGAGGACCAAAA |
|  | Reverse | CAGCCCGTATATCTTGCCGT |
| FXR | Forward | TGAGACTGGGTACCAGGGAG |
|  | Reverse | CAACACACAGCTCATCCCCT |
| HMGCR | Forward | CTTGTGGAATGCCTTGTGATTG |
|  | Reverse | AGCCGAAGCAGCACATGAT |
| HMGCS | Forward | CCAAGACTCCCTGCAACCTC |
|  | Reverse | CCAACCGTTTCCATACCCCA |
| SR-B1 | Forward | GTGCCCATCATCTGCCAACT |
|  | Reverse | TGGTGACATCAGGGACTCAGA |
| ABCG5 | Forward | CCTGCTGAGGCGAGTAACAA |
|  | Reverse | GGACGCGGAGAAGGTAGAAA |
| ABCG8 | Forward | GAAAAGAACCAGCGGGGAGA |
|  | Reverse | GCCTGGGATTTTGCCTACCT |
| LCAT | Forward | CCGTGGCTGCACTCTATGAA |
|  | Reverse | CATGGGCAGCAAATGTACGG |
| ApoA1 | Forward | GCACGTATGGCAGCAAGATG |
|  | Reverse | GGGACACATAGTCTCTGCCG |
| LXRα | Forward | TACGTCTCCATCAACCACCCC |
|  | Reverse | ACTTGCTCTGAATGGACGCTG |
| Adiponectin | Forward | GCAGAGATGGCACTCCTGGA |
|  | Reverse | CCTTCAGCTCCTGTCATTCC |
| AdipoR1 | Forward | TGGCTGATAACGGGCCATC |
|  | Reverse | GGCGTGGCTTTGTTTGTCC |
| AdipoR2 | Forward | ACTCTGACAGGATTTGGGGTC |
|  | Reverse | GTGCCCTTTTCTGAGCCGTA |
| SIRT1 | Forward | TTGGCACCGATCCTCGAAC |
|  | Reverse | CCCAGCTCCAGTCAGAACTAT |
| Irisin (FCND5) | Forward | CGAGGCTGAAAAGATGGCCT |
|  | Reverse | GCGGCAGAAGAGAGCTATAACA |
| FGF21 | Forward | GCA TAC CCC ATC CCT GAC TC |
|  | Reverse | AGG TGG GCT TCA GTG TCT TG |
| PGC1α | Forward | CCT GAA GCC GGG AGA GAA TG |
|  | Reverse | TAG CCA GCA GAG ACT GTG GA |
| ND5 | Forward | AGCATTCGGAAGCATCTTTG |
|  | Reverse | TTGTGAGGACTGGAATGCTG |
| Prdm16 | Forward | GAAGTCACAGGAGGACACGG |
|  | Reverse | CTCGCTCCTCAACACACCTC |
| Dio2 | Forward | TTGGGGTAGGGAATGTTGGC |
|  | Reverse | TCCGTTTCCTCTTTCCGGTG |
| Cidea | Forward | CTAGCACCAAAGGCTGGTTC |
|  | Reverse | CACGCAGTTCCCACACACTC |
| Elov1 | Forward | GAGAAAGGATGCCACACAAC |
|  | Reverse | GAGGCTCCATCTTTCTTTCC |
| UCP1 | Forward | CTTTGCCTCACTCAGGATTGG |
|  | Reverse | ACTGCCACACCTCCAGTCATT |
| PPARα | Forward | GTACGGTGTGTATGAAGCCATCTT |
|  | Reverse | GCCGTACGCGATCAGCAT |
| CPT1 | Forward | TGAGTGGCGTCCTCTTTGG |
|  | Reverse | TCAGCGAGTAGCGCATAGTCA |
| ACOX1 | Forward | GTGCAGCTCAGAGTCTGTCCAA |
|  | Reverse | TACTGCTGCGTCTGAAAATCCA |
| MACD | Forward | AACTAAACATGGGCCAGCGA |
|  | Reverse | GAAACCTGCTCCTTCACCGA |
| DGAT1 | Forward | TCAGATTGAGAAGCGCCTGG |
|  | Reverse | ACGGAACCCACTGGAGTGAT |
| DGAT2 | Forward | GCCGATGGGTCCAGAAGAAGTT |
|  | Reverse | CTCCAGCTTGGGGACAGTGATG |
| PPARγ | Forward | AGTGGAGACCGCCCAGG |
|  | Reverse | GCAGCAGGTTGTCTTGGATGT |
| SERBP1c | Forward | AGCAGCCCCTAGAACAAACAC |
|  | Reverse | CAGCAGTGAGTCTGCCTTGAT |
| ACC | Forward | TGACAGACTGATCGCAGAGAAAG |
|  | Reverse | TGGAGAGCCCCACACACA |
| FAS | Forward | CTGGACTCGCTCATGGGTG |
|  | Reverse | CATTTCCTGAAGTTTCCGCAG |
| GPAT | Forward | CAGACACAGGCAGGGAATCC |
|  | Reverse | GCCTAGGTCGAAATCGCGAG |
| SCD1 | Forward | TCAACTTCACCACGTTCTTCA |
|  | Reverse | CTCCCGTCTCCAGTTCTCTT |
| Arbp | Forward | TCACTGTGCCAGCTCAGAAC |
|  | Reverse | AATTTCAATGGTGCCTCTGG |
| β-actin | Forward | GGCACCACACYTTCTACAATG |
|  | Reverse | GGGGTGTTGAAGGTCTCAAAC |
